# Supplementary material for: Rodent Models for Atherosclerosis
Source: Int J Mol Sci. 2025 Dec 29;27(1):378. doi: 10.3390/ijms27010378 (PMC12786042; doi:10.3390/ijms27010378)
Supplement: Supplementary file 1 [file ijms-27-00378-s001.zip › ijms-4021471-supplementary.pdf]

Review

Rodent Models for Atherosclerosis

Linghong Zeng [1,†], Jingshu Chi [2,†], Meiqi Zhu [1], Hong Hao [2], Shiyin Long [1,*], Zhenguo Liu [2,*] and Caiping Zhang [1]

[1] Department of Biochemistry and Molecular Biology, Hengyang Medical School, University of South China, Hengyang 421001, China
[2] Division of Cardiovascular Medicine, Department of Internal Medicine, University of Nebraska Medical Center, Omaha, NE 68198, USA
* Correspondence: longshiyin@126.com (S.L.); zheliu@unmc.edu (Z.L.)
† These authors contributed equally to this work.

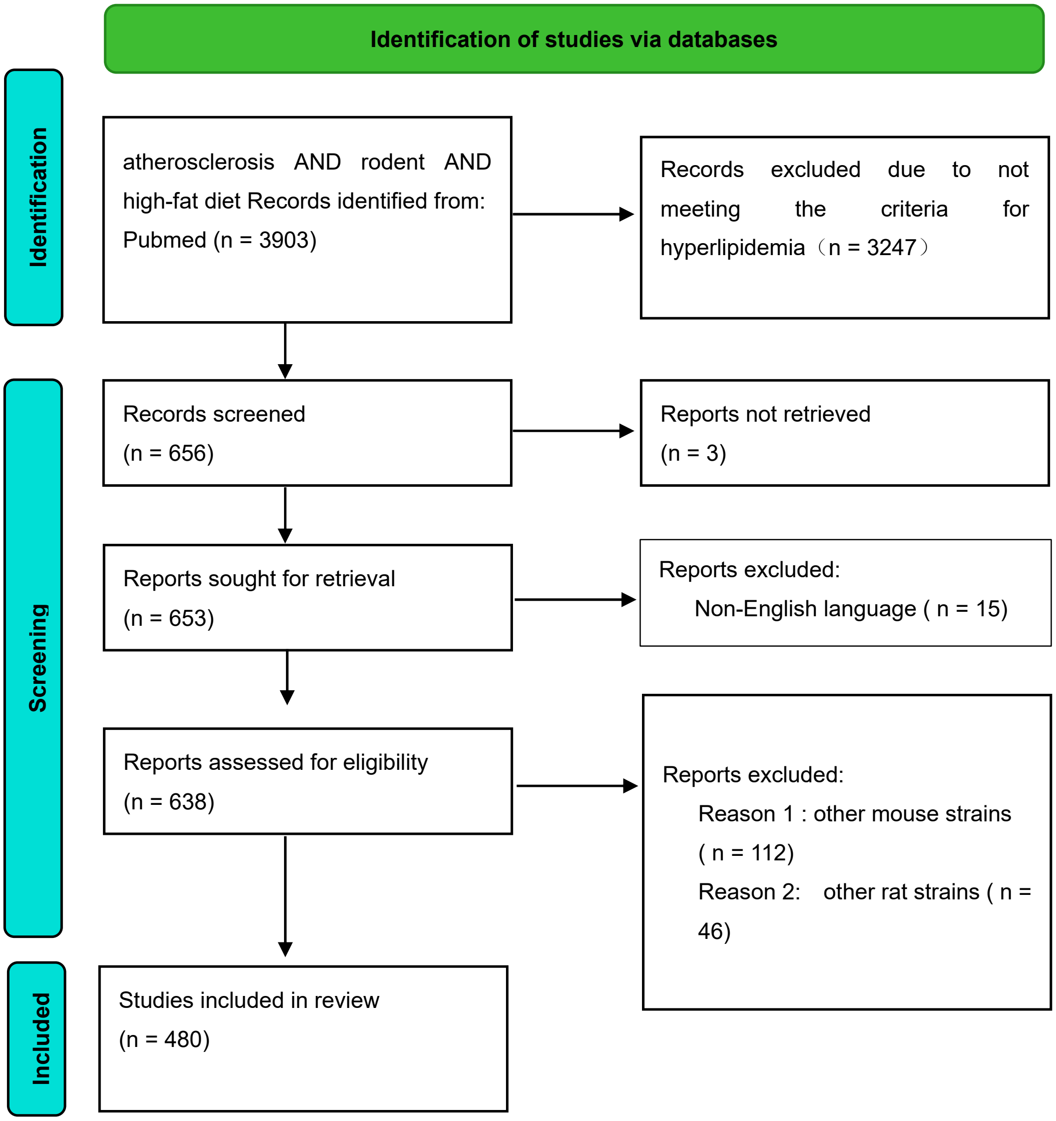

Figure S1. The flow diagram of the literature search strategy.
